# The value of high-pitch scanning with Sn100kV and ADMIRE in CT examination of tuberculous destroyed lung: Identifying the optimal combination for ultra-low-dose imaging

**DOI:** 10.1371/journal.pone.0322374

**Published:** 2025-05-05

**Authors:** Dong Jiang, Lixin Qin, Wenyang Pan, Shixiang Yan

**Affiliations:** 1 Department of Radiology, Wuhan Pulmonary Hospital, Wuhan, Hubei, China; 2 Siemens Healthineers, Shanghai, China; University of Pisa, ITALY

## Abstract

**Objective:**

To investigate the application value of high-pitch scanning combined with energy spectrum purification using Sn100kV and ADMIRE in CT examinations of patients with tuberculous destroyed lung.

**Methods:**

A total of 60 patients with sputum mycobacterium tuberculosis smear positive and diagnosed with tuberculous lung damage on imaging were prospectively collected. The first CT examination utilized a conventional scanning mode with a fixed tube voltage of 120kV, CARE Dose4D activated, reference tube current set at 70mAs, and a pitch of 1.5. The interval between the initial and follow-up CT was over three months. During the follow-up CT, a high-pitch scanning mode combined with energy spectrum purification was employed, with a fixed tube voltage of Sn100kV, CARE Dose4D activated, reference tube current set at 300mAs, and a pitch of 3.2. The remaining parameters were consistent between the two CT scans. The first CT was recorded as Group A, and the follow-up CT as Group B. After the examinations, the reconstructed layer thickness was 1.00mm, and lung window and mediastinal window images with a layer spacing of 0.7mm were obtained. The image quality of the two CT examinations was scored by three physicians using a 5-point scale. Following the scoring, the consistency of the three physicians’ scores was analyzed using the intraclass correlation coefficient.. A chief physician reviewed the lung window images from both CT scans, recorded the number of focal signs detected, and conducted Chi-square tests to compare these counts between the two groups. The CT values and noise levels in both the mediastinal window and pulmonary window were measured, SNR and CNR were calculated, and independent sample T-test was performed to analyze the differences in these parameters between the two groups. Motion artifacts in the two CT images were assessed and scored by three physicians using a 3-point values, and Mann-Whitney U test was applied to compare the scores between the groups. The radiation doses of two CT examinations was recorded, and the differences between the two groups were statistically analyzed using the Mann-Whitney U test. Data analysis was conducted using SPSS 26.0 software.

**Results:**

The image quality scores of both groups were 3 points or above, meeting the requirements for clinical diagnosis. The intraclass correlation coefficient (ICC) value for the consistency analysis of the pulmonary window scores among the three physicians was 0.819 (p < 0.001), and for the mediastinal window consistency analysis, the ICC value was 0.795 (p < 0.001), indicating good consistency in the subjective score diagnosis results. The detection rate of lesion signs in Group B was higher than that in Group A, but there was no statistical difference between the two groups (p > 0.05). There was no significant difference in noise, SNR, and CNR between the two groups (p > 0.05). However, the SNR and CNR in Group B were better than those in Group A. There was no statistical difference in the CT values of the aorta and muscle between the two groups of mediastinal window images, while noise, SNR, and CNR were statistically significant (p < 0.05). Noise in Group B was higher than that in Group A, while the SNR and CNR in Group B were lower than those in Group A. The motion artifacts of the two groups were significantly different (p < 0.001), with Group A having significantly more motion artifacts than Group B. The radiation dose of the two groups was statistically different (p < 0.001), with Group B’s radiation dose reduced by 76.24% compared to Group A.

**Conclusion:**

The combination of high-pitch scanning with Sn100kV and ADMIRE can be effectively used for ultra-low-dose CT examination of the tuberculous destroyed lung, obtaining satisfactory diagnostic images and reducing the occurrence of motion artifacts. This technique achieves conventional diagnostic outcomes at ultra-low doses and significantly reduces motion artifacts, holding significant potential and value for widespread clinical application in CT examinations for patients with tuberculous disfigured lung.

## Introduction

Tuberculous destroyed lung, a severe form of pulmonary tuberculosis, is characterized by extensive lung damage, often involving a whole lung lobe or even an entire lung side. This condition is frequently associated with extensive caseous necrosis, tuberculous cavities, and pulmonary fibrosis. It is further complicated by bronchial stenosis or dilation, mixed infections, and significant loss of lung function in advanced stages of the disease[1,2]. Managing patients with this condition is challenging due to the prolonged treatment period required. Chest X-ray examination has a low detection rate of small lung lesions [3]. Chest CT is highly valuable for diagnosing lung lesions, assessing therapeutic responses, and monitoring disease progression [4]. However, the cumulative exposure to radiation from multiple CT scans may increase the risk of cancer development. Patients with tuberculous destroyed lungs often exhibit impaired respiratory function, which can lead to difficulties in coordinating breathing during CT scans, thus increasing the likelihood of image artifacts.

High pitch mode can shorten CT scanning time and reduce motion artifacts [5], and energy spectrum purification technology tin filtration can reduce the radiation dose of CT scanning [6]. Although Sn100kV has concentrated photon energy, its penetration is limited, which easily leads to increased image noise, while iterative reconstruction can optimize image quality under low-dose CT scanning and assist in reducing radiation dose [7,8]. At present, all studies have only used energy spectrum purification or high pitch to reduce radiation dose. This paper explores the integration of high-pitch scanning with selected photon shield technology and Advanced modeled iterative reconstruction (ADMIRE) algorithm, leveraging the technical capabilities of Siemens’ third-generation dual-source CT scanner. The aim is to achieve ultra-low dose CT scanning in patients with tuberculous destroyed lung, ensuring the maintenance of high-quality lung imaging without compromising diagnostic accuracy.

## Materials and methods

### Patient characteristics

From June 1, 2022, to October 31, 2023, patients with positive mycobacterium tuberculosis smears in sputum of our hospital and diagnosed with destroyed lung during their first CT examination were prospectively collected and included in the study. The first CT examination used a conventional scanning protocol and was scored as the Conventional Group (Group A). If the patient required a follow-up CT examination due to their condition, a spectral purification combined with high-pitch and ADMIRE scanning protocol was used during the follow-up CT, and the patients were classified into the High-Pitch Group (Group B). A total of 60 cases meeting the study criteria were statistically analyzed, including 32 males and 28 females, with an average age of 42.52 ± 10.21 years. The duration of tuberculosis was more than 3 years for all patients; the duration of anti-tuberculosis treatment ranged from 20 to 55 months, with a median time of 32 months. The clinical data of cases are shown in Table 1. This study was approved by the Ethics Committee of the hospital.

**Table 1:** Clinical data sheet for included cases.

Inclusion criteria	Exclusion criteria	Clinical diagnostic data of included cases
BMI < 25 kg/ m2	Presence of thoracic malformations	60 cases were clinically diagnosed with secondary pulmonary tuberculosis
Age ranging from 20 to 65 years old	Pregnancy, including women who are pregnant or breastfeeding	40 with drug-resistant pulmonary tuberculosis
Sputum smear positive for mycobacterium tuberculosis and diagnosed with destroyed lung through radiographic imaging	Patients with metal implants in the chest cavity	12 with tuberculous pleurisy
		8 with bronchial tuberculosis
Sputum smear positive for mycobacterium tuberculosis and diagnosed with destroyed lung through radiographic imaging	Have a history of chest surgery	18 with pulmonary tuberculosis combined with infection
The weight change between the two CT examinations was less than 2 kg		45 with pulmonary tuberculosis combined with cavitation

Note: Each patient had one or more clinical findings.

### Dual-source computed tomography techniques

Patients were examined using a third-generation dual-source CT system (Somatom Force; Siemens Healthcare, Forchheim, Germany). They were positioned supine with their hands holding their heads. For the standard group (group A), a fixed tube voltage of 120kV was used, with CARE Dose4D activated, a reference tube current of 70mAs, and a pitch of 1.5. For the high pitch group (group B), a fixed tube voltage of Sn100kV was used, with CARE Dose4D activated, a reference tube current of 300mAs, and a pitch of 3.2, and other parameters were kept consistent with Group A. The reconstruction algorithm used for both groups was ADMIRE with strength level of 5. Thin slice reconstruction was performed with a slice thickness of 1.0 mm and an interslice gap of 0.7 mm for both groups. Standard lung reconstruction algorithms were employed for both groups, along with a convolution kernel of Br57 for lung windows and Br40 for mediastinal windows. The scanning range extended from the lung apex to 1 cm below the costophrenic angle for both groups.

### Image analysis

Image quality was assessed using a 5-point scale, employing a double-blind method for evaluating the lung and mediastinal windows. This evaluation was conducted by three senior diagnostic physicians: 1 point: The image is non-diagnostic; it cannot be used for clinical assessment due to severe limitations; 2 points: The anatomical structures are poorly defined, and the image is marred by significant artifacts, which hinder a clear diagnosis; 3 points: The anatomical structures are recognizable, and while artifacts are present, the image quality is sufficient for diagnosis when combined with multi-directional reconstruction techniques; 4 points: The anatomical structure of the tissue is clear, with slight artifacts; 5 points: The anatomical structures are clearly depicted without any artifacts, representing optimal image quality. A score of 3 or higher is considered adequate for satisfying diagnostic requirements. After the completion of the score, the results of the three doctors were analyzed for consistency.

Group A and B images were reviewed by a chief physician in a double-blind manner. Ten characteristic signs were examined on the thin lung window images, and the number of signs found was recorded. These signs included: Tree bud sign, Pulmonary cavity, Pulmonary consolidation, Pulmonary ground-glass shadow, Tractive bronchiectasis, Nodule shadow, Mosaic sign and air retention, Emphysema, Interlobular septal thickening, Cellular lung.

A supervisor technician performed double-blind scanning of thin mediastinal window images. CT values and standard deviation (SD) of aorta and vertical spinal muscle were measured at the level of the bronchial bifurcation. Signal-to- noise rations (SNR) and contrast-noise ratio (CNR) were calculated using the following notations: the CT value of the aorta was denoted as CT1, noise as SD1, and the CT value of the spinal muscle was denoted as CT2, noise as SD2, SNR = CT1/SD1, CNR= (CT1-CT2)/SD1.

A technician, using a double-blind method, measured the noise and CT values of lung parenchyma and trachea at three consecutive levels at the tracheal bifurcation on thin lung window images. The average of the three measurements was taken as the final result. The CT value of lung parenchyma was denoted as CT3, and the noise was denoted as SD3. The average value of the three points was taken as the final result. During the measurement, SNR1, SNR1 = CT3/SD3 were calculated, and the CT value and noise of the trachea at the same level were measured. The CT value and noise of the trachea were denoted as CT4 and SD4, and CNR1, CNR1=(CT3-CT4)/SD4 were calculated.

Respiratory movement artifacts and cardiac pulsation artifacts were scored separately by the above three diagnostic doctors using a double-blind method. In cases of differing scores, a 3-point system was adopted through negotiation: 3 points for no artifacts, 2 points for mild artifacts, and 1 point for obvious artifacts.

The CT volume dose index (CTDIvol (mGy)), dose length product (DLP (mGy.cm)), and effective dose (ED(mSv)) generated by the CT machine were recorded. The effective dose was calculated using the formula ED = DLP × 0.014mSv/ (mGy.cm) [9].

### Statistical methods

The statistical analysis was performed using SPSS 26.0 software. For continuous data that met the assumptions of normality and homogeneity of the variance, comparisons between the two groups were conducted using an independent sample t-test, with results expressed as mean ± standard deviation. For non-normally distributed continuous data, the Mann-Whitney U test was used, with results presented as median and interquartile range [M (IQR)]. Categorical data were analyzed using the Chi-square test, with results described as frequency (rate). The intra-class correlation coefficient (ICC) was used to assess the consistency of the diagnostic results among the three physicians. In all bilateral tests, a significance level of less than 0.05 was considered statistically significant.

## Results

**Fig 1:**
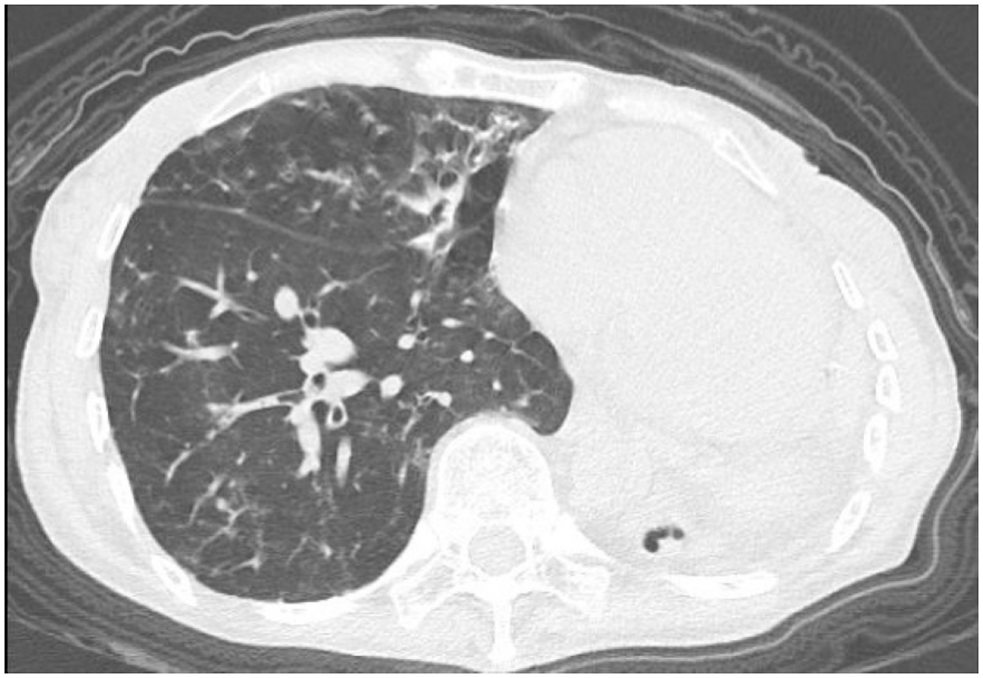
Lung Window (120 kV Scan). The right lung exhibits scattered patchy opacities, nodules, tree bud signs, and dot stripe patterns. Faint bronchial columnar changes are observed. Obvious respiratory movement artifacts are present. The volume of the left lung appears reduced.

**Fig 2:**
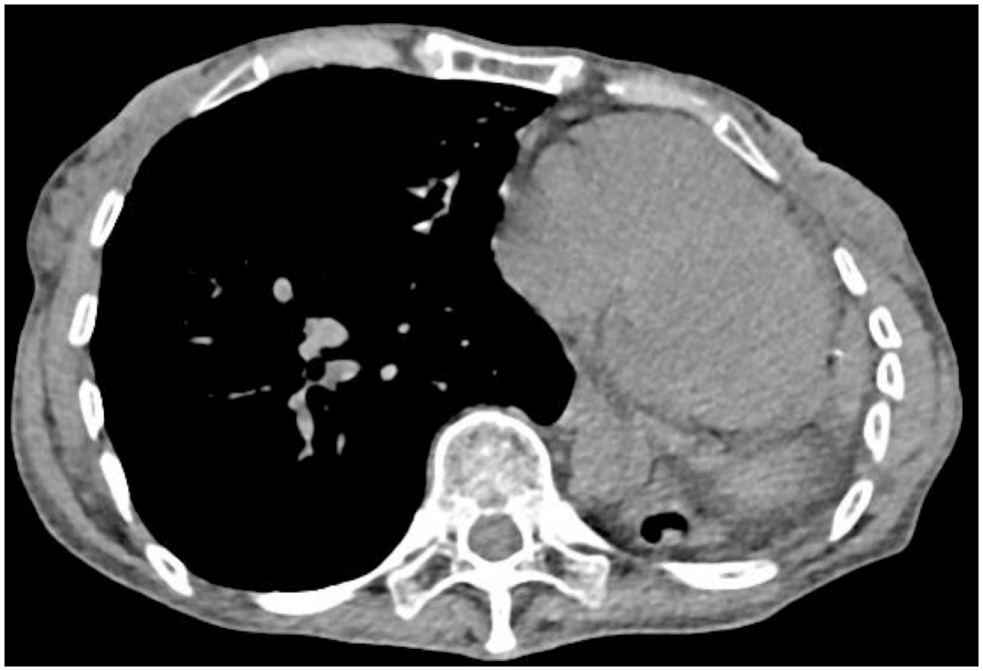
Mediastinal Window (120 kV Scan). The left lung shows soft tissue density and a translucent area indicative of insect erosion. The left side of the heart and surrounding structures are visualized.

**Fig 3:**
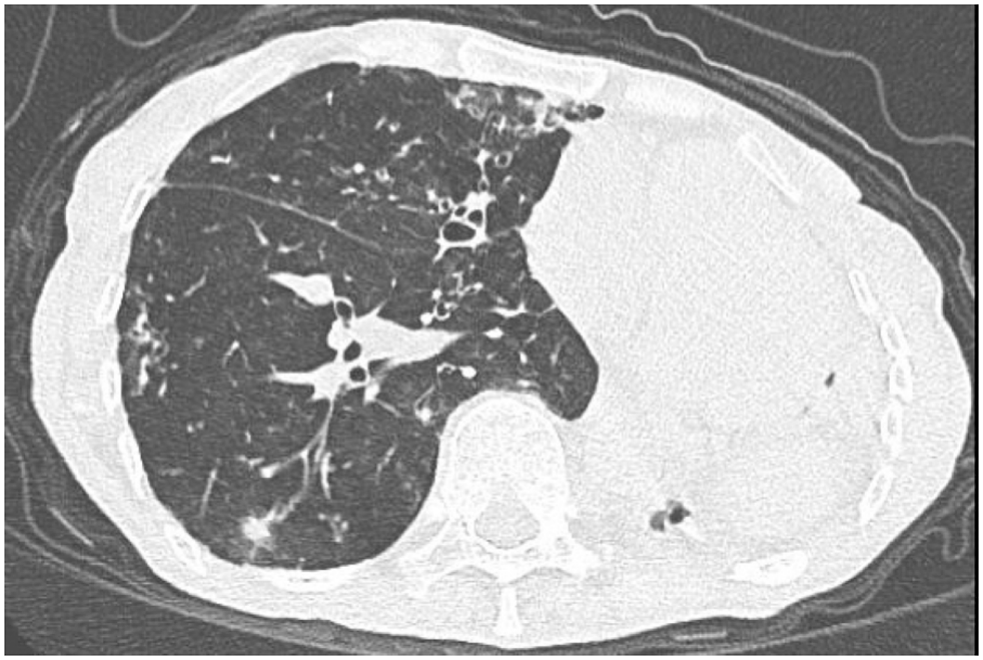
Lung window (Sn100kV scan). Multiple scattered patchy opacities, small nodules, tree-in-bud opacities, and heterogeneous dot-like/linear densities are observed in the right lung. The lesion morphology remains consistent with that shown in Fig 1. Compared to Fig 1, bronchiectatic changes are distinctly visible within the bronchial architecture, and no significant respiratory motion artifacts are observed.

**Fig 4:**
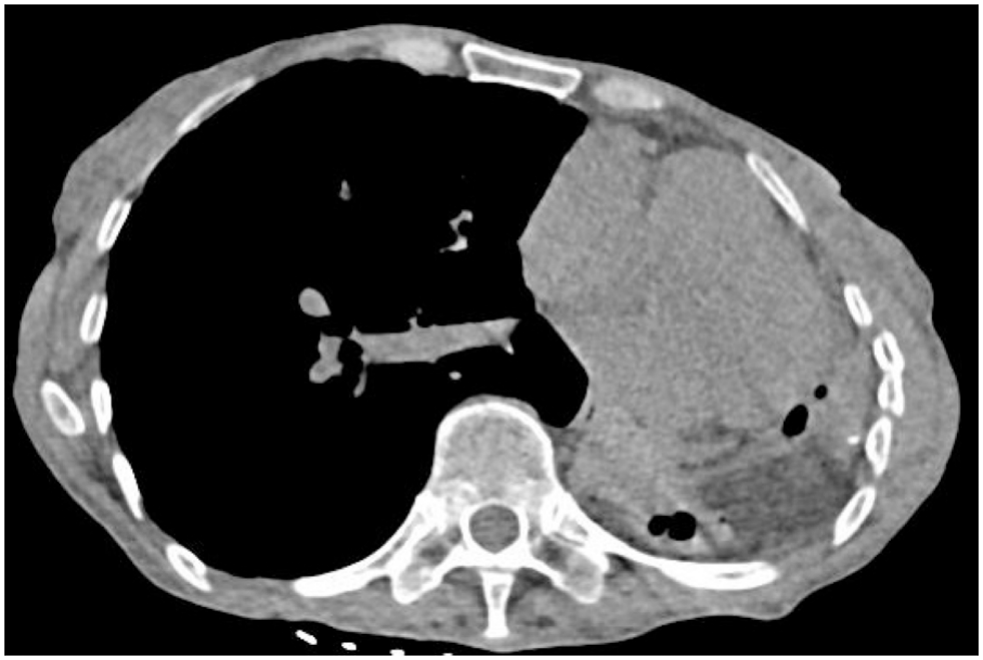
Mediastinal windows (Sn100kV scan). Leftward cardiac displacement is observed. The left lung demonstrates a soft-tissue density lesion containing moth-eaten lucent area. Image qquality remains comparable to that of Fig 2 in terms of anatomical delineation and artifact control.

The ICC was used to assess the consistency of the diagnosis results among the three physicians, with all scores being 3 points or above. The results indicated that the ICC value for the pulmonary window score diagnostic test was 0.819 (95%CI 0.762-0.866) p < 0.001), and the reproducibility was good. The ICC value of the mediastinal window score diagnostic test was 0.795 (95%CI 0.733-0.847) (p < 0.001), and the reproducibility was good. (Table 2). The image quality under conventional conditions is shown in Fig 1 and 2, while the image quality with the high pitch and selected photon shield technology is shown in Figs 3 and 4.

**Table 2:** Three physicians’ subjective ratings of Group A and Group B images.

Grouping	Lung window score					Mediastinal window score				
**1**	**2**	**3**	**4**	**5**	**1**	**2**	**3**	**4**	**5**
**Radiologist 1**										
**A**	0	0	8	38	14	0	0	10	46	4
**B**	0	0	0	15	45	0	0	18	37	5
**Radiologist 2**										
**A**	0	0	6	38	16	0	0	11	42	7
**B**	0	0	0	8	52	0	0	10	47	3
**Radiologist 3**										
**A**	0	0	10	40	10	0	0	9	50	1
**B**	0	0	0	13	47	0	0	19	38	3

Fig 1–4. Comparative CT Imaging of a 60-Year-Old Female with a 4-Year History of Tuberculosis, Bronchiectasis, Cavity, and Left Lung Destruction.

The number of detections of tree bud sign, pulmonary ground glass shadow, Mosaic sign, air retention, emphysema, interlobular thickening and cellular lung in group B was higher than that in group A. However, no statistical differences were observed between the two groups (p < 0.05), as shown in Table 3.

**Table 3:** Comparison table of pathological signs detected in Groups A and B.

Pathological characteristics	Group A	Group B	χ2	p
**Tree bud sign**	43(10.59%)	45(10.71%)	0.003	0.954
**Pulmonary cavity**	67(16.5%)	67(15.95%)	0.046	0.830
**Pulmonary consolidation**	54(13.3%)	54(12.86%)	0.036	0.850
**Pulmonary ground-glass shadow**	30(7.39%)	34(8.1%)	0.144	0.704
**Tractive bronchiectasis**	40(9.85%)	40(9.52%)	0.025	0.873
**Nodule shadow**	35(8.62%)	35(8.33%)	0.022	0.882
**Mosaic sign and air retention**	34(8.37%)	37(8.81%)	0.05	0.823
**Emphysema**	40(9.85%)	41(9.76%)	0.002	0.965
**Interlobular thickening**	37(9.11%)	39(9.29%)	0.007	0.932
**Cellular lung**	26(6.4%)	28(6.67%)	0.023	0.879

**Fig 5:**
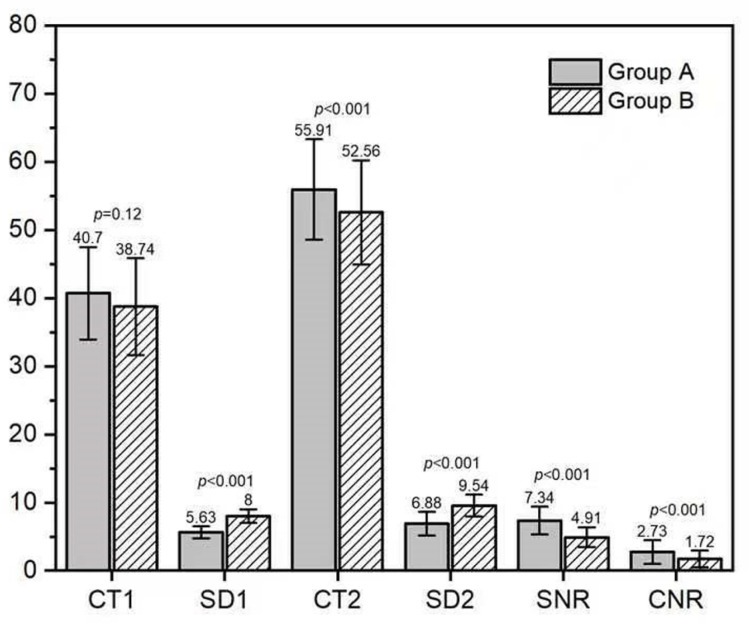
Objective evaluation of mediastinal window in group A and group B images.

**Fig 6:**
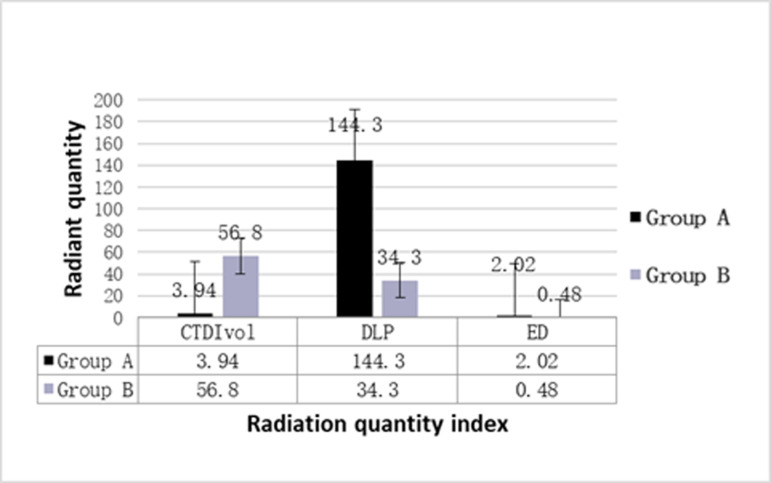
Comparison of radiation doses between group A and group B.

There were no significant differences in CT values of aorta and muscle between the two groups. However, significant differences were noted in noise, SNR and CNR between the two groups (p < 0.05). Group B exhibited higher noise levels and lower SNR and CNR compared to Group A, as shown in Fig 5. There were no statistical differences in the evaluation indices of the lung window between the two groups (p > 0.05). However, Group B demonstrated better SNR1 and CNR1 values for the lung window compared to Group A, as presented in Table 4. The scores for respiratory movement artifacts were 3.0 (2.0-3.0) in group A and 3.0 (3.0-3.0) in group B, indicating fewer respiratory movement artifacts in Group B (Z = 4.388, p < 0.001). The cardiac pulsation artifacts were 2.0 (2.0-2.0) in Group A and 3.0 (2.25-3.0) in Group B, with Group B showing fewer artifacts (Z = 8.027, p < 0.001), as detailed in Table 5. Both DLP and ED were lower in group B compared to group A. The effective dose in Group B was reduced by 76.24% relative to Group A, with statistically significant differences between the two groups (p < 0.001), as summarized in Fig 6.

**Table 4:** Table of objective evaluation results of lung window in group A and group B.

Grouping	CT3	SD3	CT4	SD4	SNR1	CNR1
**A group**	-888.75 ± 35.34	18.79 ± 3.9	-976.35 ± 8.58	17.92 ± 4.34	-49.75 ± 12.64	5.25 ± 2.60
**B group**	-885.9 ± 34.62	18.17 ± 3.91	-975.93 ± 8.56	17.18 ± 4.26	-51.34 ± 12.79	5.64 ± 2.72
**Z value**	0.364	-0.713	0.222	-0.762	-0.557	0.663
**p-value**	0.717	0.478	0.825	0.449	0.579	0.509

**Table 5:** Statistical table of motion artifacts in group A and group B.

Index	Grouping	Score			Z Value	p-value
		**3**	**2**	**1**		
**Respiration** **Movement**	A	39	21	0	4.388	<0.001
	B	58	2	0		
**Cardiac** **Pulsation**	A	2	49	9	8.027	<0.001
	B	45	15	0		

## Discussion

Current methods to reduce radiation dose and optimize image quality in CT scans include reducing tube voltage, reducing tube current, increasing pitch, reducing the number of scans, and using iterative reconstruction techniques. Akmal Sabarudin et al. [10] confirmed that the tube voltage is proportional to the square of the radiation dose, and reducing the tube voltage can effectively reduce the radiation exposure. Deng Jiantao et al. [11] compared 120kV and 100kV in the Adamkiewicz Artery and found that the radiation dose of Sn100kV, purified by energy spectrum was reduced by 76.24% (0.48mSv vs 2.02mSv) compared to 120kV. The energy spectrum purification technology (Sn100kV) filters low-energy rays by placing a tin filter plate in front of the X-ray tube, improving ray efficiency and thus reducing the radiation dose [12]. This technology is particularly effective in reducing radiation dose in this study. There is a positive correlation between the tube voltage and noise, and the image noise will increase when the tube voltage is reduced. Energy spectrum purification technology offers two modes: Sn150 kV and Sn100 kV. Sn150 kV has strong penetration and is recommended for abdominal applications [13], while Sn100kV has limited penetration and is most recommended for lung applications [14]. In this study, objective evaluation of mediastinal window showed that Group B had higher noise and lower SNR and CNR compared to Group A, while noise, SNR1, and CNR1 were similar in the lung window. This discrepancy may be due to two factors: First, lung tissue has good contrast with surrounding structures, making it less susceptible to noise, resulting in consistent measurements in the lung window. However, increased noise can reduce tissue contrast in the mediastinum, leading to different measurements in the mediastinal window. Second, energy spectrum purification retains only high-energy rays, improving ray utilization. Image quality scoring by three physicians showed that both groups scored 3 points or higher, indicating that Group B images meet clinical diagnostic requirements and that Sn100 kV is feasible for lung applications. This finding aligns with previous research [15], suggesting that image quality comparable to conventional CT scans can be achieved.

The relationship between the tube current and radiation dose is linear, while the image noise is inversely proportional to the tube current. When other scanning conditions are consistent, a higher tube current results in a higher radiation dose and lower image noise. In this study, Group B had a lower tube voltage than Group A, which would typically lead to increased image noise. However, by setting the reference tube current of Group B four times higher than that of Group A, the noise in Group B was only 3 HU higher than in Group A, a difference that is imperceptible to the human eye (Table 3). This indicates that the higher tube current in Group B effectively optimizes image noise, having no impact on diagnostic outcomes. Furthermore, ADMIRE was applied in this study. ADMIRE optimizes image noise under low tube voltage through multiple iterations of noise reduction technology, thereby assisting in reducing the radiation dose [16–18]. The combination of higher tube current and ADMIRE ensures that Group B maintains a very low noise value even at a low tube voltage. Park Min Su et al. [19] demonstrated that the application of advanced iterative algorithms and high-level current modulation at low tube voltage can reduce radiation dose and improve image quality, findings that are consistent with those of this study.

Theoretically, the radiation dose of Group B should increase due to the higher tube current. However, the radiation dose of Group B was lower than that of Group A, as shown in Fig 6. This finding aligns with the research of Jijo Paul et al. [20], suggesting that the impact of tube current on radiation dose is less significant than that of tube voltage. Additionally, Group B utilized a lower tube voltage and high pitch technology. Previous studies [21,22] have confirmed that high-pitch scanning can significantly reduce radiation dose compared to conventional spiral scanning. The low tube voltage in Group B, combined with advanced iterative reconstruction and high -pitch technology, offsets the increased radiation dose due to the higher tube current, resulting in a final radiation dose for Group B that is still below 0.5 mSv. These results underscore the significant value of the Group B scanning protocol in reducing radiation dose and optimizing image quality.

Patients with tuberculous pulmonary destruction often have impaired lung function, making it difficult for them to hold their breath during CT scanning. High-pitch scanning technology offers a solution by completing whole-lung scanning in sub-millisecond levels, minimizing the impact of breathing and heartbeat on image quality. Niraj Nirmal Pandey et al. [23] confirmed that high-pitch scanning can significantly reduce motion artifacts, improving the detection rate of subtle lesion signs in the pulmonary window. In this study, the entire lung was scanned in approximately 0.35 seconds using high-pitch technology, compared to about 4 seconds with conventional spiral scanning. The shorter scanning time not only reduces the radiation dose but also minimizes motion artifacts. This finding is consistent with the research results of Joshua G Hunter et al. [24], further validating the superiority of high-pitch scanning in reducing motion artifacts and enhancing diagnostic accuracy.

This study verifies the clinical advantages of combining energy spectrum purification technology (Sn100kV), high pitch and ADMIRE in patients with tuberculous destroyed lung. in patients with tuberculous destroyed lung. Compared to conventional CT scanning mode, this integrated technique not only provides satisfactory diagnostic images but also significantly reduces the radiation dose, minimizes motion artifacts, and enhances the detection rate of subtle lesion signs. By ensuring high-quality images while reducing radiation exposure, this technique holds significant potential and broad applicability in CT examinations for patients with tuberculous lung disfigurement.

## Conclusions

The combination of high pitch scanning, with Sn100kV and ADMIRE can be effectively utilized for ultra-low dose CT examination in patients with tuberculous destroyed lung. This approach not only yields satisfactory diagnostic images but also significantly reduces the occurrence of motion artifacts, thereby enhancing the diagnostic accuracy and safety of the procedure.

## Abbreviations

ADMIRE: Advanced modeled iterative reconstruction
BMI: Body mass index
CTDIvol: Volume computed tomography dose index
DLP: Dose length product
ED: Effective dose
SD: Standard deviation.
